# Editorial: How entrepreneurs' identity influences their wellbeing in entrepreneurial process

**DOI:** 10.3389/fpsyg.2022.1121066

**Published:** 2023-01-05

**Authors:** Song Lin, Shubo Liu, Wencang Zhou

**Affiliations:** ^1^Strategy Department, Central University of Finance and Economics, Beijing, China; ^2^Management Department, Montclair State University, Montclair, NJ, United States

**Keywords:** entrepreneurship, identity, wellbeing, framework, research domain

Identity research focuses on how an entrepreneur answers the question of “who I am” or “who I want to be.” Researchers find that entrepreneurs' identity has a significant influence on entrepreneurs' individual characteristics, such as passion, persistence, and self-efficacy. Evidence also shows that identity can influence entrepreneurs' psychological status such as wellbeing. O'Neil et al. (2022) suggested that if entrepreneurs are not able to assure their own identity, it would harm their wellbeing. Entrepreneurial wellbeing refers to the experience of satisfaction, positive affect, infrequent negative affect, and psychological functioning in the relation to developing, starting, growing, and running an entrepreneurial venture.

Entrepreneurs' wellbeing is important not only to a firm's performance and survival, but also to entrepreneurs' opportunity recognition and exploration, which are the core dimensions of entrepreneurship. Therefore, we argue that the relationship between entrepreneurs' identity and their psychological status is a promising topic in the fields of both entrepreneurship and psychology and that explaining this relationship in theory and data is an urgent task for researchers. In this Research Topic, we aim to gain a better understanding of how and why entrepreneurs' identity influences their wellbeing during the entrepreneurial process. It is noteworthy that, during the COVID-19 crisis, entrepreneurs are facing enormous pressure. Further research about identity and wellbeing can be beneficial for entrepreneurs to keep a healthier psychological status in a hostile environment.

The selected articles in this issue are all about enterpreneurs' identity and their wellbeing. Some of these articles are a direct study of these two concepts.

Yang et al. proposes a double-edged sword model of the effect of entrepreneurial identity on subjective wellbeing. Results suggest that on the one hand, entrepreneurial identity induces entrepreneurs' work-related affective rumination to reduce their subjective wellbeing through the path of resource depletion, on the other hand, entrepreneurial identity stimulates entrepreneurs' contemplation on work-related problem-solving pondering to enhance their subjective wellbeing through the path of resource acquisition. In the path of resource depletion, work-related affective rumination produces a “suppressing effect” between an entrepreneur's identity and entrepreneurial subjective wellbeing. In addition, entrepreneurial mindfulness weakens the resource depletion path.

Chen et al. investigates the relationship between entrepreneurs' Darwinian social identity and business performance *via* CSR. The empirical results indicate that entrepreneurs' Darwinian social identity contributes positively to CSR, so as further to business performance. In addition, this relationship is further found to be significantly moderated by entrepreneurs' wellbeing. The results indicate that entrepreneurs can achieve business success *via* CSR, by which entrepreneurs can further acquire successful entrepreneurship through caring more about their wellbeing.

Xu and Jia focuses on the influence of COVID-19 on entrepreneurs' psychological wellbeing (PWB) in China. Based on conservation of resources (COR) theory, this study found that COVID-19 will significantly decrease entrepreneurs' PWB. A start-up's past performance will enhance the negative influence of COVID-19 on entrepreneurs' PWB. This study contributes to the literature on entrepreneurship, COR, and PWB. The findings can also guide entrepreneurs to maintain wellbeing during the pandemic and post-pandemic era.

Other articles do not directly study identity or wellbeing, but their research content is also highly relevant to this topic.

Guo et al. introduces the process of the deepened rigidity in WS Co. Company, which occurs due to the wrong cognition of Dr. S and his teams who managed or failed to respond to the rigidity encountered by the company during different periods. The rigidity of its capacity is believed to be caused by the cognitive errors of the entrepreneur and his team facing every period of potential rigidity, which makes them fail to deal with it (or choose the wrong methods), thus leading to complete rigidity.

Zafar et al. focuses on a specific type of entrepreneurial behavior, that is, social entrepreneurship (SE). By assessing a sample of 810 employees from active enterprises, this paper discovers that social performance mediates positively and partially between social entrepreneurship orientation and financial performance, and both direct and indirect paths are in the same direction and significant. The study adds contribution to the literature, which has not been testified before on hybrid firms, and gives the researchers/scholars new directions to address related disciplines and further explore this domain.

Zhou and Li analyzes the influence of farming experience on urban residents' entrepreneurial decisions, while a mediating effect model was used to test its channels of action. The results show that: farming experience can contribute to the entrepreneurial decision of urban residents relative to those without experience in farming. Heterogeneity tests based on age, city type, and physical capital found that this effect was more significant in urban residents with non-capital cities, middle-aged groups, and high-material capital. Farming experience indirectly drives entrepreneurial decisions through the mediating role of promoting positive personality traits, such as “optimism” and “mutual aid consciousness.”

Based on a single-case study in Q Village, Jiang, Ma, et al. identifies the key role played by relational contracts in entrepreneurship groups. Primary Action Group transforms exploratory intuitive learning into exploratory compilation learning, and Secondary Action Group triggers the learning effect and makes a proprietary investment by utilizing intuitive formulaic learning and compiled formulaic learning, thus reducing unforeseen, contracting and verification costs. During the pattern maturity period, Primary Action Group rationally integrates the supply chain and forms a stable entrepreneurial paradigm, while Secondary Action Group does so to maintain prior information reserves and lower information search, supervised execution, and bargaining decision costs.

Jiang, Wu, et al. investigates the influence of clan networks and farmers' entrepreneurial income. Based on the social capital theory, we adopt a semilogarithmic model, and propensity score matching method for robustness checks. The results show that the clan network, as an endowed social capital of farmer, has a significant and positive effect on entrepreneurial income for both men and women. And the clan network has the greatest impact on middle-income farmers.

The collection of these articles provides a positive reference for how to conduct research in the field of Entrepreneurs' identity and their wellbeing. In the future, more diversified qualitative or quantitative research is required in this field, so as to increase more theoretical insights.

## Author contributions

As the editors of this Research Topic, SLin, SLiu, and WZ participated in the planning of the Research Topic, the solicitation and review of articles, and made comments on the contributions of each article. All authors contributed to the article and approved the submitted version.
